# A new genus and species of arboreal toad with phytotelmonous larvae, from the Andaman Islands, India (Lissamphibia, Anura, Bufonidae)

**DOI:** 10.3897/zookeys.555.6522

**Published:** 2016-01-20

**Authors:** S. R. Chandramouli, Karthikeyan Vasudevan, S. Harikrishnan, Sushil Kumar Dutta, S. Jegath Janani, Richa Sharma, Indraneil Das, Ramesh K. Aggarwal

**Affiliations:** 1 Wildlife Institute of India, Chandrabani, Dehradun-248001, Uttarakhand, India; 2 Centre for Ecological Sciences, Indian Institute of Science, Bangalore, India; 3 CSIR-CCMB, Laboratory for the Conservation of Endangered Species, Pillar 162, PVNR Expressway, Hyderguda, Attapur Ring Road, Hyderabad 500048, India; 4 Nature Environment and Wildlife Society (NEWS), Nature House, Gaudasahi, Angul, Odisha, India; 5 Centre for Cellular and Molecular Sciences (CSIR-CCMB), Uppal Road, Tarnaka, Hyderabad, 500007, India; 6 Institute of Biodiversity and Environmental Conservation, Universiti Malaysia Sarawak, 94300 Kota Samarahan, Kuching, Sarawak, Malaysia

**Keywords:** Amphibian, bufonid, tadpole, rRNA, molecular phylogeny, skeletal characters

## Abstract

A new bufonid amphibian, belonging to a new monotypic genus, is described from the Andaman Islands, in the Bay of Bengal, Republic of India, based on unique external morphological and skeletal characters which are compared with those of known Oriental and other relevant bufonid genera. *Blythophryne* **gen. n.** is distinguished from other bufonid genera by its small adult size (mean SVL 24.02 mm), the presence of six presacral vertebrae, an absence of coccygeal expansions, presence of an elongated pair of parotoid glands, expanded discs at digit tips and phytotelmonous tadpoles that lack oral denticles. The taxonomic and phylogenetic position of the new taxon (that we named as *Blythophryne beryet* **gen.** et **sp. n.**) was ascertained by comparing its 12S and 16S partial genes with those of Oriental and other relevant bufonid lineages. Resulting molecular phylogeny supports the erection of a novel monotypic genus for this lineage from the Andaman Islands of India.

## Introduction

Neobatrachian anurans of the family Bufonidae Gray, 1845 are represented in the Oriental portion of Asia by 14 genera (Table 1). Recent analyses of both morphological and molecular data have revealed that several terrestrial genera such as *Adenomus*, *Duttaphrynus*, *Ingerophrynus* and *Xanthophryne* had remained obscurely hidden under the catch-all generic name ‘*Bufo*’ Garsault, 1754 (*fide* Manamendra-Arachchi and Pethiyagoda 1998; Frost et al. 2006; Biju et al. 2009). Likewise, the arboreal forms of Oriental Asia were once considered to be members of the African genus *Nectophryne* Buchholz & Peters, 1875 (see Boulenger 1892, 1896, 1919), till Barbour (1938) recognised the morphological variations and allocated them to two different genera by revalidating Günther’s (1875) *Pedostibes* and describing as new *Pelophryne*. Following this taxonomic treatment, subsequent studies on the systematics of Oriental arboreal toads have reconfirmed the distinctiveness of these genera and have led to the recognition of additional bufonid genera, based on morphological as well as molecular evidence (see Fei et al. 2003; Matsui et al. 2007).

**Table 1.** Members of the Neobatrachian anurans of the family Bufonidae Gray, 1845 represented in the Oriental portion of Asia.

	Genus	Number of species	Distribution
1	*Adenomus* Cope, 1860	2	Sri Lanka
2	*Ansonia* Stoliczka, 1870	28	Sundaland and Philippine archipelago
3	*Bufoides* Pillai & Yazdani, 1973	1	Khasi Hills, Meghalaya, India
4	*Duttaphrynus* Frost, Grant, Faivovich, Bain, Haas, Haddad, de Sá, Channing, Wilkinson, Donnellan, Raxworthy, Campbell, Blotto, Moler, Drewes, Nussbaum, Lynch, Green & Wheeler, 2006	29	Eastern Africa to Papua New Guinea; 25 species are known from India and south east Asia
5	*Ingerophrynus* Frost, Grant, Faivovich, Bain, Haas, Haddad, de Sá, Channing, Wilkinson, Donnellan, Raxworthy, Campbell, Blotto, Moler, Drewes, Nussbaum, Lynch, Green & Wheeler, 2006	12	Indochina and the Sundaland
6	*Leptophryne* Fitzinger, 1843	2	Sundaland
7	*Parapelophryne* Fei, Ye & Jiang, 2006	1	Indochina
8	*Pedostibes* Günther, 1876 “1875”	5	Western Ghats and Eastern Himalayas, India; Malay Peninsula, Borneo & Sumatra
9	*Pelophryne* Barbour, 1938	11	Sundaland and the Philippines Archipelago
10	*Phrynoidis* Fitzinger, 1843	2	Indochina-Sundaland
11	*Pseudobufo* Tschudi, 1838	1	Sundaland
12	*Sabahphrynus* Matsui, Yambun & Sudin, 2007	1	Borneo
13	*Xanthophryne* Biju, Van Bocxlaer, Giri, Loader & Bossuyt, 2009	2	Northern Western Ghats, India
14	*Ghatophryne* Biju, Bocxlaer, Giri, Loader & Bossuyt, 2009	2	Western Ghats, India

Of all the above, *Duttaphrynus melanostictus* (Schneider, 1799) is the only bufonid reported from the Andaman Islands (Sarkar 1990; Das 1999). In the adjacent Nicobar archipelago; however, a second putative taxon, *Docidophryne spinipes* (a *nomen nudum*) was reported earlier (Fitzinger 1861), which was subsequently described erroneously as a new taxon, *Bufo camortensis* by Mansukhani and Sarkar (1980) from Camorta, in the central Nicobar Islands. Both these were later synonymised with *Bufo melanostictus* by Crombie (1986). Recent herpetological surveys conducted in the Andaman Islands resulted in the collection of a diminutive, arboreal toad species in the hill forests of Mt. Harriet National Park and on a few adjacent islands, which is described herein, allocated to a new monotypic genus and compared with other currently valid (Frost 2014) Oriental and related bufonid genera.

## Materials and methods

### Specimen collection and preservation

Specimens were hand-collected, euthanised and fixed in absolute ethanol for a minimum of 24 hours, and eventually transferred to 60% ethanol for preservation. Tissue samples were extracted and stored in absolute ethanol (prior to specimen fixation) for phylogenetic analyses. Tadpoles were collected and reared for preservation of samples across developmental stages in 4% formalin solution. Conspecificity between tadpoles and the adults was confirmed by rearing them to metamorphosis, as well as matching 16S ribosomal DNA sequences to those of the adults. Staging of tadpoles follow Gosner (1960). Type specimens were deposited in the collection of the Zoological Survey of India, Kolkata (ZSIC). Museum abbreviations follow Sabaj Pérez (2012) except for WII, which represents vertebrate collections at the Wildlife Institute of India, Dehradun, India. Morphometric measurements were done using Mitutoyo™ dial calipers, to the nearest 0.01 mm, between 3-4 months of preservation of the adults and tadpoles. Morphometric measurements (Table 2) were recored for all the adults, metamorphs, and tadpoles.

Skeletal characters of a paratype were examined under a microscope by clearing using trypsin and 0.5% potassium hydroxide solution and staining with alcian blue and alizarin red dye, following Hanken and Wassersug (1981). Additional information on osteology of selected genera for comparison is based on an examination of comparative material (Appendix I), as well as published literature. Webbing formulae follow Savage and Heyer (1997). Geographic coordinates of the localities were recorded using a Garmin GPSmap 78s (map datum WGS84). Calls were recorded using a digital stereo microphone and analysed using Raven™ and are archived in the Macaulay Library, Cornell Lab of Ornithology (Voucher no: ML 174095).

Tadpoles were described based on collections made in May 2011, from a phytotelm, located *ca*. 1.3 m above the ground. The clutch was monitored continuously till complete transformation. The observed eggs got transformed into pale white embryos on 2 May 2011; subsequently, tadpoles at different developmental stages were collected and preserved in 5% formalin. Tail tips of these individuals were collected and preserved in absolute ethanol for DNA barcoding studies before the tadpoles were preserved in formalin.

**Table 2.** Abbreviations and definitions of morphometric measurements made on adult, metamorph of frogs and tadpole. Measurements made only on tadpoles are indicated by an asterisk after the abbreviation.

**AG**	Distance from posterior point of the forelimb at its insertion into the body to the anterior point of the hindlimb insertion
BL*	Distance from snout tip to the point of the initiation of tail from the body
**BW**	Distance at the broadest point at the trunk
DFH*	Height of the fin measured at the place of the maximum height of the dorsal fin
**DL fold**	Length of dorso-lateral fold
**ED**	Horizontal diameter of the orbit
**EN**	Distance between anterior border of the eye to posterior edge of the nostril
**ES**	Distance between anterior border of the eye to the snout tip
**ETY**	Distance between posterior border of the eye to anterior margin of the tympanum
**f1 to 4**	Distance measured from the fork of the fingers to the tip of the finger disc for fingers 1 to 4
**FEL**	Distance measured from the cloaca to the tip of the knee
**FOL**	Distance measured from the anterior end of the tarsus to the tip of the fourth toe
**HD**	Height of the head measured at the post-orbital region before the parotoid gland
**HL**	Distance from the tip of the snout to the posterior edge of the mandible
**HW**	Width of the head measured at the jaw angle
**IN**	Closest distance between the nares
IND*	Distance between the external nares
**IO**	Distance between the anterior margins of the upper eyelids
IOL*	Distance between the two orbits
**LAL**	Distance measured from the elbow to the base of the outer metacarpal tubercle; palm length
MBW*	Distance measured at point of the maximum width of the body
MTH*	Distance measured at the point of the maximum height of the tail by laterally positing the tadpole
MTMW*	Distance measured on the tail at the point of initiation of the tail from the body where the tail width is maximum
**NA**	Not measured
NED*	Distance between nostril and eye
NSD*	Distance from the snout to the eye
ODD*	Oral disc diameter
**PAL**	Distance measured from the posterior border of the outer metacarpal tubercle to the tip of the third finger
**PL**	Length of the parotoid gland
**PW**	Maximum width of the parotoid gland
SS*	Distance from snout to the spiracle
SV*	Distance from snout to the vent
**SVL**	Distance from tip of the snout till the cloaca
**t1 to 5**	Distance measured from the fork of the toe to the tip of the toe disc for toes 1 to 5
**TBL**	Distance from the knee to the obtuse margin of the tibia
TL*	Distance from the point of initiation of tail till the tip of the tail
TMH*	Distance measured on the tail at the point where the tail muscle reaches maximum height
**TYH**	Horizontal diameter of the tympanum
**TYV**	Vertical diameter of the tympanum
**UAL**	Distance measured from the point of insertion of the forelimb to the trunk to elbow
**UEW**	Maximum width of the upper eyelid
VFH*	Ventral fin height measured at the place of the maximum height of the ventral fin
VTL*	Vent tube length

**Molecular phylogeny.** Total genomic DNA was extracted from the alcohol-preserved soft tissue (muscle), taken from the holotype, following the standard procedure of SDS & proteinase-K lysis, followed by chloroform-isoamyl extraction method. The taxonomic position of the toad was ascertained by rDNA typing of both 16S and 12S rDNA genes of the mitochondrial genome broadly following the method as described earlier by Dutta et al. (2004). The parts of 16S and 12S rDNA were amplified and sequenced for both strands using the published primers (Palumbi et al. 1991), 16Sar-L [5´-CGCCTGTTTATCAAAAACAT-3´], 16Sbr-H [5´-CCGGTCTGAACTCAGATCACGT-3´] and 12saL [5´-AAACTGGGATTAGATACCCCACTAT-3´], 12sbH [5´-GAGGGTGACGGGCGGTGTGT-3´], respectively. The raw sequences from both strands were end-clipped, edited and assembled to build partial 12S (417 bp) and 16S (551bp) gene sequences of the taxon individually. The sequences were subjected to BLAST search against the NCBI database sequences in order to ascertain the gene and broad taxonomic identity. Multiple sequence alignments using CLUSTALX 2.0 (Thompson et al. 1997), along with representative Asian and African origin sequence homologs under the Bufonidae, spanning 21 genera and 43 species (Table 3), were constructed individually for both 12S and 16S partial genes. Subsequently, manually edited alignments of both 12S and 16S were concatenated to get a final single alignment, which was then used for all further phylogenetic analysis. Initially, the analysis was conducted using sequence data of 36 species of the 21 genera and *Rhaebo guttatus* as outgroup to ascertain the broad affinity of the new taxon in the Bufonidae. Subsequently, sub-trees were constructed using mainly the Asian toad species and *Ghatophryne*, *Pedostibes*, and *Adenomus* as successive outgroups, to better resolve the phylogenetic status of the new taxon.

**Table 3.** Taxon sampling for phylogenetic analysis of selected Oriental members of the Bufonidae.

Taxon	Range/ Collection location	NCBI Acc. No.	Tree_7a	Subtree_7b	Subtree_7c	Reference
*Blythophryne beryet* gen. et sp. n.	India (A&N Islands)	KT991336, KT991347	+	+	+	This study
*Adenomus kelaartii*	Sri Lanka	FJ882780	+	+	+	Bocxlaer et al. 2009
*Amietophrynus brauni*	Tanzana	DQ158437	+			Pramuk et al. 2008
*Amietophrynus gracilipes*	Equatorial Guinea	DQ158456	+			Pramuk et al. 2008
*Amietophrynus gutturalis*	Kenya	DQ158460	+			Pramuk et al. 2008
*Amietophrynus poweri*	Namibia	DQ158482	+			Pramuk et al. 2008
*Amietophrynus steindachneri*	Kenya	DQ158488	+			Pramuk et al. 2008
*Ansonia hanitschi*	Malaysia	FJ882794	+			Bocxlaer et al. 2009
*Ansonia longidigita*	Malaysia	KT991329, KT991340	+			This study
*Bufo bufo*	Turkey	DQ158438	+			Pramuk et al. 2008
*Bufoides meghalayanus*	India	KT991331, KT991342	+	+	+	This study
*Duttaphrynus atukoralei*	India	FJ882835		+	+	Bocxlaer et al. 2009
*Duttaphrynus brevirostris*	India	FJ882786		+	+	Bocxlaer et al. 2009
*Duttaphrynus crocus*	India	FJ882789		+	+	Bocxlaer et al. 2009
*Duttaphrynus dhufarensis*	India	FJ882837		+	+	Bocxlaer et al. 2009
*Duttaphrynus himalayanus*	India	KT991334, KT991345	+	+	+	This study
*Duttaphrynus hololius*	India	FJ882781		+	+	Bocxlaer et al. 2009
*Duttaphrynus melanostictus*	India	KT991335, KT991346	+	+	+	This study
*Duttaphrynus parietalis*	India	FJ882784		+	+	Bocxlaer et al. 2009
*Duttaphrynus scaber*	India	KT991332, KT991343	+	+	+	This study
*Duttaphrynus stomaticus*	India	KT991333, KT991344	+	+	+	This study
*Duttaphrynus stuarti*	India	FJ882788		+	+	Bocxlaer et al. 2009
*Ghatophryne ornata*	India	FJ882797	+	+		Bocxlaer et al. 2009
*Ingerophrynus divergens*	Malaysia	KT991328, KT991339	+			This study
*Ingerophrynus galeatus*	Laos	DQ158452	+			Pramuk et al. 2008
*Ingerophrynus macrotis*	Laos	DQ158468	+			Pramuk et al. 2008
*Leptophryne borbonica*	Malaysia	FJ882799	+			Bocxlaer et al. 2009
*Mertensophryne micranotis*	Tanzania	FJ882821	+			Bocxlaer et al. 2009
*Mertensophryne uzunguensis*	Tanzania	FJ882819	+			Bocxlaer et al. 2009
*Nectophryne afra*	Cameroon	DQ283360	+			Frost et al. 2006
*Nectophryne batesi*	Gabon	DQ283169	+			Frost et al. 2006
*Nectophrynoides minutus*	Tanzania	FJ882814	+			Bocxlaer et al. 2009
*Nectophrynoides tornieri*	Tanzania	DQ283413	+			Frost et al. 2006
*Pedostibes hosii*	Malaysia	KT991330, KT991341	+			This study
*Pedostibes tuberculosus*	India	FJ882793	+	+		Bocxlaer et al. 2009
*Pelophryne api*	Malaysia	KT991326, KT991337	+			This study
*Phrynoidis asper*	Brunei	DQ158431	+			Pramuk et al. 2008
*Phrynoidis juxtasper*	Malaysia	KT991327, KT9913387	+			This study
*Sabahphrynus maculatus*	Malaysia	AB331718	+			Matsui et al. 2007
*Schismaderma carens*	Zimbabwe	DQ158424	+			Pramuk et al. 2008
*Vandijkophrynus robinsoni*	Namibia	GU183857	+			Bocxlaer et al. 2010
*Xanthophryne koynayensis*	India	FJ882782	+	+	+	Bocxlaer et al. 2009
*Rhaebo guttatus*	Brazil	DQ158459	+			Pramuk et al. 2008

For each of the phylogenetic analysis, the concatenated 12S+16S sequence alignment was first used to find the best fitting DNA substitution model using Akaike Information criterion (AIC), as implemented in jModelTest2 (Guindon and Gascuel 2003; Darriba et al. 2012) was found to be for both the domains. Phylogenetic analysis was then conducted using the inferred GTR+G+I base substitution model and both Maximum likelihood (ML) and Bayesian inference (BI) methods. BI was implemented in MrBayes 3.1.2 (Ronquist and Huelsenbeck 2003) using the following parameters: GTR+G+I model of DNA substitution, Nst as 6 (all different substitution rates subjected to GTR), flat substitution rates and the stationary nucleotide frequencies of the GTR rate matrix (as calculated using Dirichlet Process Prior; Heath et al. 2012), a uniform distribution (0,1) for both, the shape parameter of the gamma distribution of rate variation and the prior for the proportion of invariable sites; 3,000,000 MCMC iterations in two runs and four chains; with sampling every 300 iterations; minimum standard deviation of the split frequencies as 0.01; burn-in of initial 25% of stored trees and parameters. Similarly, ML analyses were implemented in RaxML (Stamatakis 2006) with 500 replicates, applying a separate GTRGAMMA model to each partition. The output tree was visualised using Figtree (http://tree.bio.ed.ac.uk/software/figtree/). For comparison based on genetic distances, uncorrected k2p pair-wise distances were calculated both within and across genus for both 16S and 12S partial gene sequences in MEGA 6.06 (Tamura et al. 2013), using the complete deletion option.

## Results

### Systematics

#### Blythophryne gen. n.

Taxon classificationAnimaliaORDOFAMILIA

http://zoobank.org/2BAB0154-53B8-43E3-BB14-F36F12FDD8DE

##### Type species.

**Figure 1.**
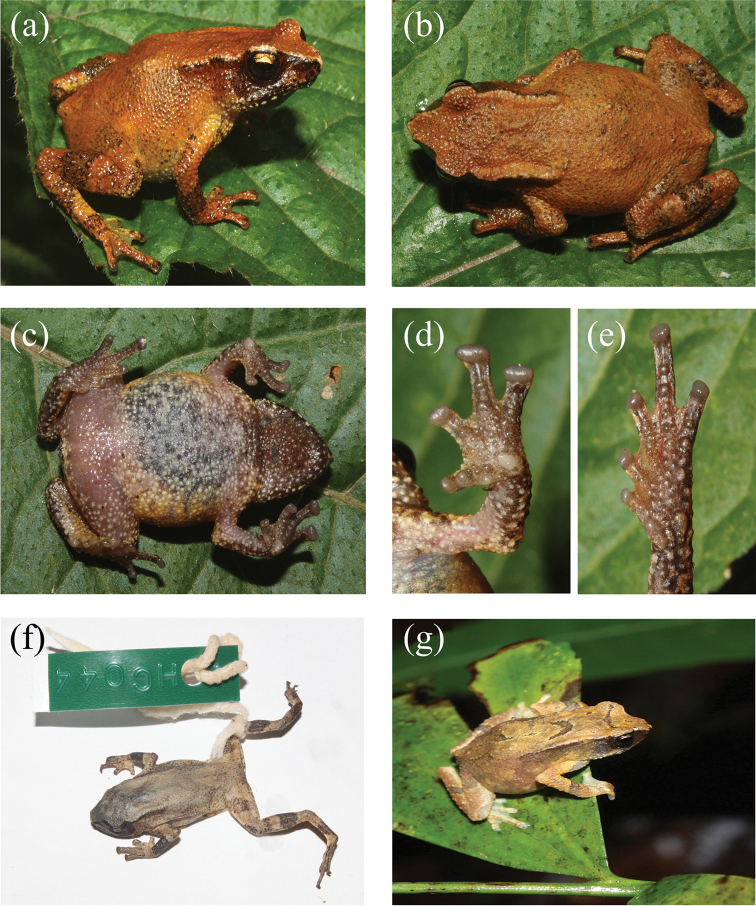
Morphological characters of the *Blythophryne beryet* gen. et sp. n.: **a** dorso-lateral view **b** dorsal view **c** ventral view **d** ventral view of left palm **e** ventral view of left foot of the adult female holotype (ZSI_A-12521) in life **f** adult female holotype in preservation **g** dorsal view of the male paratype (ZSI_A-12529) in life showing inverted-V shaped markings and the inter-ocular band on the dorsum.

*Blythophryne beryet* gen. et sp. n. by monotypy (Fig. 1, Table 4).

**Table 4.** Morphometric measurements of the holotype and paratype series of adult and two gravid (g) individuals of *Blythophryne beryet* gen. et sp. n.

	ZSI A-12521	ZSI A-12524	ZSI A-12522	ZSI A-12523	ZSI A-12526	ZSI A-12529	ZSI A-12527	ZSI A-12530	ZSI A-12528	ZSI A-12525
**Sex**	♀	♀(g)	♂	♀(g)	♂	♂	♀	♂	♂	♂
**SVL**	27.4	25.5	25.5	25.2	24.5	23.0	22.7	22.3	22.2	21.8
**AG**	10.6	9.2	9.8	12.5	8.0	7.3	8.0	6.7	6.5	8.5
**HL**	7.7	7.5	8.2	6.9	7.5	7.9	7.5	7.6	7.6	7.1
**HW**	7.9	7.6	8.1	6.8	8.0	7.8	7.6	7.7	7.4	7.2
**HD**	4.3	3.5	3.9	3.4	3.9	3.4	3.2	3.2	3.0	3.2
**BW**	9.9	10.3	9.1	11.8	8.3	7.1	6.1	7.5	6.3	9.8
**EN**	2.2	2.3	1.7	1.9	1.9	2.3	2.1	2.0	2.1	2.2
**ES**	3.4	3.4	3.3	3.1	3.5	3.3	3.2	3.2	3.3	3.1
**ETY**	0.5	0.7	0.7	0.7	0.7	0.7	0.7	0.6	0.6	0.5
**UEW**	1.9	1.5	2.0	1.8	1.8	1.7	1.7	1.5	1.9	1.8
**IO**	3.8	3.8	3.5	3.4	3.5	3.8	3.4	3.5	3.6	3.4
**IN**	2.2	2.1	2.2	2.2	1.8	2.1	2.1	1.9	2.0	1.9
**TYH**	1.6	1.6	1.9	1.6	1.6	1.4	1.5	1.4	1.8	1.5
**TYV**	1.8	1.7	1.9	1.6	1.6	1.6	1.6	1.5	1.8	1.5
**UAL**	5.1	4.7	4.4	5.2	4.3	4.3	4.3	4.6	4.3	4.1
**LAL**	5.8	5.4	5.6	5.5	5.6	5.5	5.4	5.3	5.4	5.3
**PAL**	6.2	5.7	6.2	6.8	5.8	5.9	5.9	5.8	5.9	6.1
**FEL**	9.2	7.5	7.7	7.2	9.3	8.2	9.5	8.2	8.6	8.5
**TBL**	10.6	8.0	9.4	8.4	9.1	7.9	9.0	8.1	8.3	8.5
**FOL**	9.6	9.7	9.4	8.3	9.4	8.0	9.2	8.3	8.7	8.3
**ED**	2.8	2.5	2.4	2.6	2.5	2.1	1.9	2.4	2.1	2.3
**DL fold**	13.3	12.2	11.3	11.9	12.0	12.3	11.7	12.0	11.4	11.9
**PL**	6.1	5.9	6.5	6.0	4.5	3.7	4.0	5.9	3.2	3.9
**PW**	1.4	1.4	1.3	1.6	1.0	1.0	0.9	1.3	0.9	0.9
**f1**	1.8	1.2	1.1	1.5	1.6	1.3	2.0	1.2	1.6	0.9
**f2**	1.9	1.4	1.6	1.7	2.2	1.8	2.2	1.4	1.9	1.6
**f3**	3.1	3.0	2.9	2.8	2.8	2.6	2.8	2.9	2.4	2.9
**f4**	2.2	1.9	1.8	2.1	2.2	1.9	2.1	1.8	1.6	1.8
**t1**	1.1	1.1	1.2	1.1	1.3	1.0	1.3	1.2	1.0	1.1
**t2**	1.4	1.7	1.4	1.4	1.7	1.4	1.5	1.1	1.5	1.5
**t3**	2.6	2.0	2.0	2.7	2.3	2.1	2.6	2.1	2.2	1.9
**t4**	4.7	4.1	3.9	4.9	4.4	2.9	4.6	3.7	3.0	4.0
**t5**	3.0	2.1	2.3	2.6	2.5	2.1	2.5	2.1	2.0	1.9

##### Content.

A single species is currently known.

##### Type material.

Holotype ♀ : ZSI_A-12521(Fig. 1), (SVL 27.4 mm) leg. S. R. Chandramouli and S. Harikrishnan on 12 December 2010 near Mt. Harriet National Park (ca. 11°42'N, 92°44'E, 175 m asl.) within evergreen forests at *ca*. 2130 hours. Paratypes (paratopotypes): ZSI_A-12522 to ZSI_A-12530 (three ♀ and six ♂; Fig. 1g); leg. S. R. Chandramouli and S. Harikrishnan during 22 - 25 June 2010 from the above location but at an altitude range of ~150–330 m asl. Other paratypes (larvae): seven tadpoles (WII-115) collected from a phytotelm on Rutland Island. Referred material: WII-113, an adult topotype with six toes on both the feet.

##### Etymology.

The generic name is a patronym, coined in appreciation of Edward Blyth (1810–1873), the first curator of the Asiatic Society of Bengal, who initiated herpetological studies in the Andaman and Nicobar Islands, through his phenomenal, pioneering paper “Notes on the fauna of the Nicobar islands” (Blyth 1846). Das (1999) remarked, “Blyth is to be credited for the description of a large number of species from the Andaman and Nicobar Islands that are still valid. Blyth (1846) wrote the first account on the vertebrate fauna of these islands, and in 1863, compiled the first check-list”. Further details of Edward Blyth and his contributions to studies on Indian natural history are in Das (2004) and Sridharan (2013). The specific epithet ‘*beryet*’ (in Great Andamanese language; http://www.andamanese.net/Great_Andamanese_Lexicon_English.pdf) refers to ‘small frog’. We believe that the Great Andamanese knew of the existence of this small arboreal anuran that is here described as new species to science. We hope the name given here will also raise awareness about the dwindling, indigenous tribal populations in the Andamans, their culture and extinction of their tribal languages.

##### Diagnosis.

This currently monotypic genus and species is diagnosed by the following suite of external morphological and osteological characters: small adult size (mean SVL 24.0 mm; range 21.8–27.4 mm); distinct tympanum, slightly smaller than eye; absence of cephalic ridges; absence of vomerine teeth; presence of a single, median, external vocal sac in males; presence of elongated pair of parotoid glands; absence of enlarged, keratinised tubercles on dorsum; presence of well developed, sheath-like webbing on fingers and on toes; digit tips dilated to discs, lacking circum-marginal grooves; presence of six presacral vertebrae; urostyle lacking lateral dilations; absence of omosternum and presence of arciferal pectoral girdle. Mature ova small (0.62 mm mean diameter), yolky and unpigmented; tadpoles lacking keratodont.

##### Description of the holotype.

A small bufonid (mean SVL 24.2 ± 0.6 mm), with depressed, moderately robust (AG:BW 1.0) habitus (Fig. 1a–c). Head almost as long as broad (HL:HW 0.97), devoid of cephalic ridges, with a single, median internal vocal sac in males. Snout obtusely pointed in dorsal view, projecting beyond mandibles; nostrils oriented laterally, situated on lateral fold closer to tip of snout than to eye (EN:ES 0.7), loreal region mildly concave, canthal ridge well defined between nostril and the eye, distance between orbit and nostril greater than internarial distance (IN:EN 0.96), upper eyelid rough, densely covered with minute warts, eyes large (ED:HL 0.4), about twice length of tympanum (TYH:ED 0.6), separated from each other by twice internarial distance (IN:IO 0.6), and over twice width of upper eyelid (IO:UEW 1.9), pineal ocellus absent; vomerine teeth absent, tongue elongate, slender and oval, free posteriorly, not bifid, lacking lingual papilla; dorsolateral fold conspicuous, almost up to 48% SVL, beyond which it becomes indistinct and disappears; parotoid glands slender and elongate (PL:PW 4.3), as well-defined postorbital ridge. Limbs slender, upper arm short, 18.7% of SVL, lower arm longer than the upper arm (21% SVL), fingers basally webbed, webbing between Fingers II and III not exceeding penultimate subarticular tubercle (webbing formula I_0-1_II_1-2_III_2-1_IV; Fig. 1d); an enlarged, prominent outer metacarpal tubercle at palmar base (subequal to disc on Finger I), nuptial pad absent , subarticular tubercles prominent on fingers and toes, finger tips dilated to discs lacking circummarginal grooves that are much broader than long, and are less discernible in the first and second fingers; relative length of fingers 3 > 4 > 2 > 1; thigh 33.7% SVL, subequal to shank (38.6% SVL); toes partially webbed, webbing between Toes III and IV extending to penultimate subarticular tubercle (webbing formula I_0-1_II_0-1_III_1-2_IV_2½-½_V; Fig. 1e); tarsal ridge absent, inner meta-tarsal tubercle larger than outer. Relative length of toes 4 > 5 > 3 > 2 > 1. Skin rough dorsally and granular ventrally; lower abdomen with free, loose skin flap. Tubercles or granules absent on dorsum, scattered over venter, under surface of thighs less granular; throat and limb-insertions with dense granules, tibia with enlarged granular tubercles.

##### Colouration in life.

Dorsum reddish-brown, with two feeble dark brown inverted ‘V’ shaped markings which fail to reach flanks, interorbital band indistinct, canthus dark chocolate brown, colour extending a little beyond tympanum, subequal to half-length of parotoid gland; forearm and hind limbs barred, one each on thigh, shank and tarsus. Venter heavily speckled with dark brown spots, throat dark brown, lower lip spotted with white and brown, pupil large, horizontally elliptical.

##### Colouration in alcohol.

Dorsum drab brown with indistinct ‘inverted-V’ shaped pattern, darker bands on limbs, venter cream, with black mottled pattern, throat black throughout (Fig. 1f).

##### Osteology

**Figure 2.**
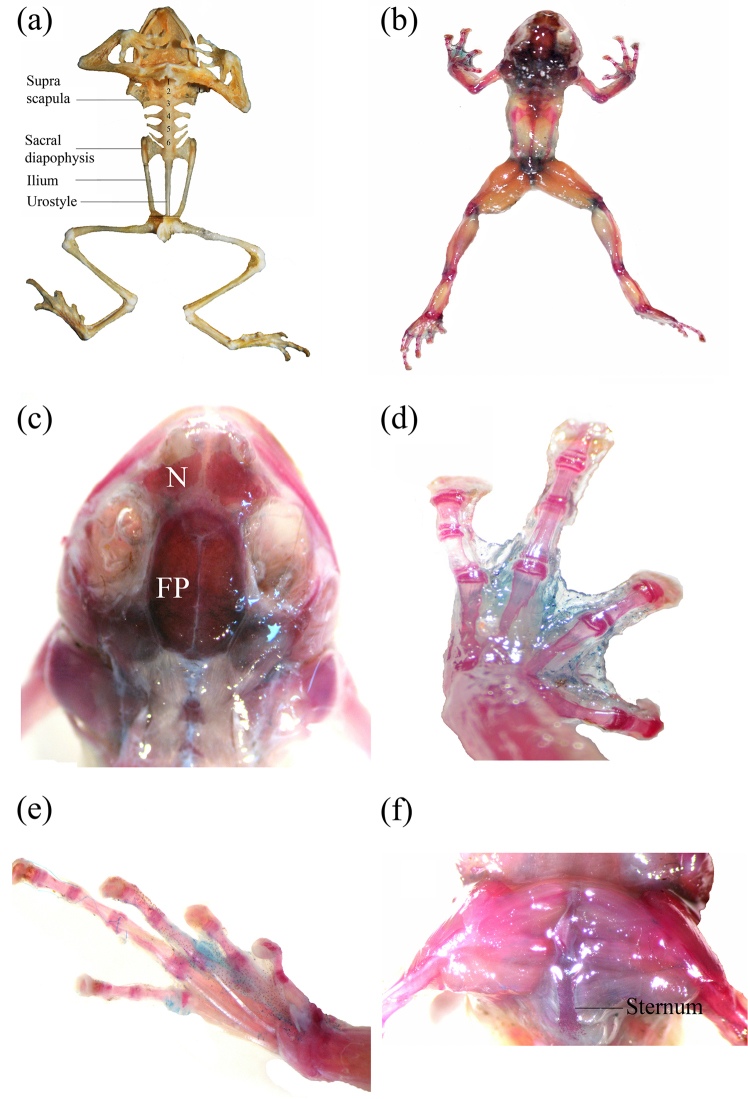
Skeletal characters of paratypes (ZSI_A-12527) of *Blythophryne beryet* gen. et sp. n. **a** complete dry structure **b–f** various characters visible after staining/clearing of the skeleton. FP – frontoparietal; N – nasal.

(based on paratype ZSI_A12527). Axial and appendicular skeleton composed primarily of bony elements; cartilaginous elements not observed. Atlas (the first vertebra) with rudimentary hypapophysis and not fused to axis, presacral vertebrae six in number, Vertebrae II–V bearing horizontally elongate hypapophyses, those on Vertebrae II and V oriented anteriorly; Vertebrae III–IV oriented horizontally; sacral diapophysis laterally dilated; coccyx not fused to sacrum; articulating with former by a double condyle and lacking lateral expansions, omosternum absent, pectoral girdle arciferal, with epicoracoids united to each other anteriorly and overlapping posteriorly (Fig. 2). Phalangeal formula of fingers 2-2-3-3; toes 2-2-3-4-3, terminal phalange obtusely curved, not truncate. Nasal bones of the skull large, about 1/3^rd^ of frontoparietals and 1.25 times as large as orbital cavity. Maxillary and vomerine teeth absent.

##### Morphological variations.

Adult females and males range between 25.2–27.4 mm and 21.8–25.5 mm, respectively. Measurements of paratypes are provided in Table 4. Dorsal colour in different shades of brown or reddish-brown. Intensity of inverted ‘V’-shaped pattern on dorsum variable. On one occasion, an abnormal specimen (WII-113) with a deformity was observed, with six digits, the first toe being preceded by a small additional toe on both feet. Fingers showed no such anomalies.

##### Description of calls.

**Figure 3.**
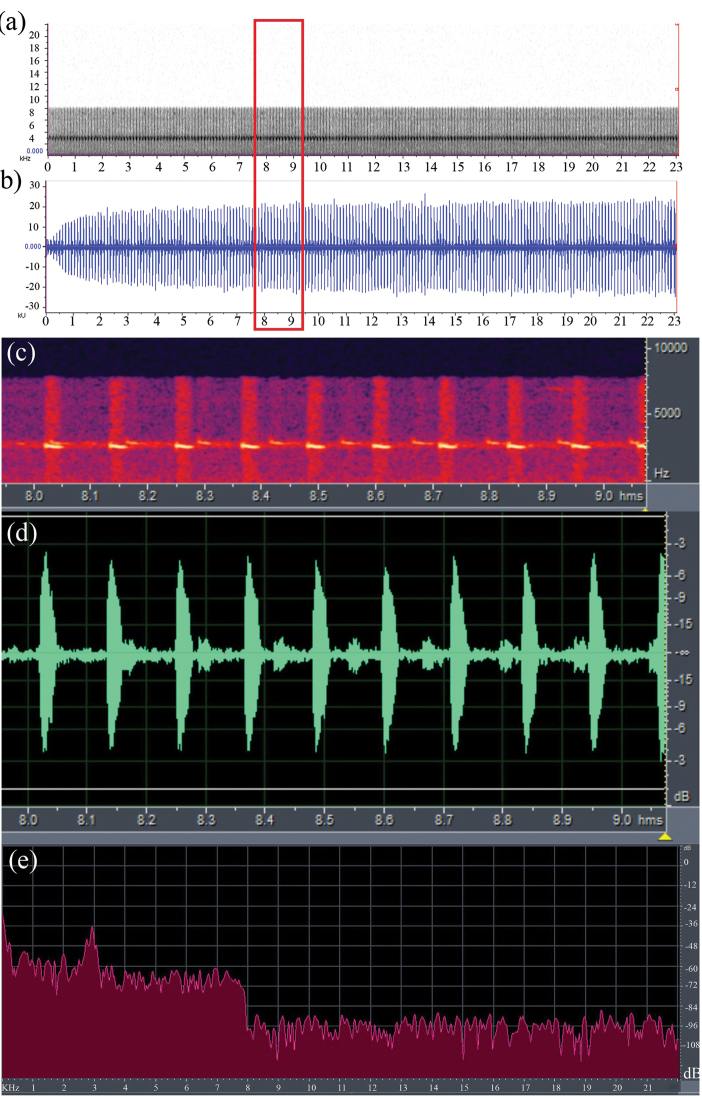
Sound spectrogram **a** and oscillogram **b** of a 23 second clip of a call of *Blythophryne beryet* gen. et sp. n. . Detailed view of **c** frequency and **d** amplitude modulations of a one second long clip of the call **e** power spectrum of the call of *Blythophryne beryet* gen. et sp. n.

(Macaulay Library, Cornell Lab of Ornithology; voucher no: ML 174095). A calling male was observed on 24 November 2010 on the surface of leaves within bushes. Calls were composed of continuous syllables of “pip-pip-pip-pip-pip-” at a constant frequency of 8 kHz, without pause, lasting for 23 seconds, with mean amplitude of -3 db / 20 kU (Fig. 3). The call was composed of 198 pulses uttered within duration of 23 s, at a rate of 8 to 9 (mean = 8.6) pulses per second. Each pulse lasted for duration of 0.3 s (n = 198) with an interval of 8.5 s (n = 197) between two consecutive pulses.

##### Distribution.

**Figure 4.**
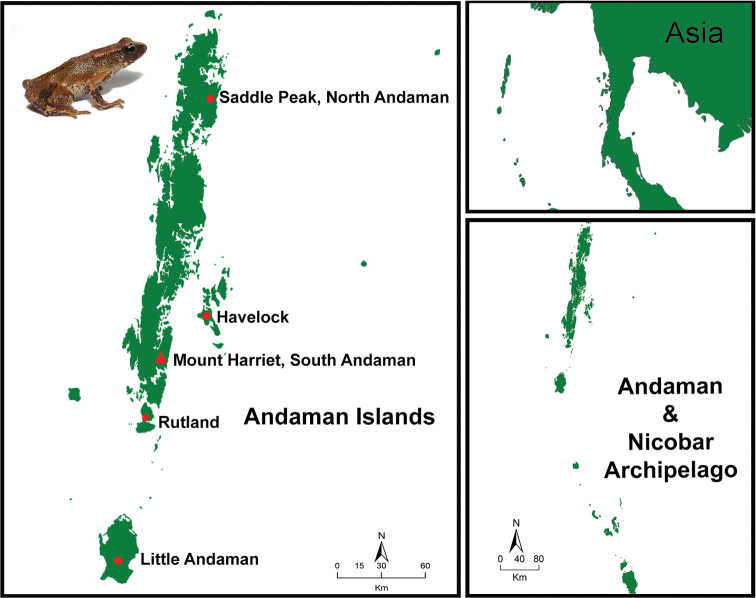
Map showing distribution of *Blythophryne beryet* gen. et sp. n. in the Andaman Islands, Bay of Bengal, India. Holotype collected from Mt Harriet (indicated with a red triangle).

This species has been documented from five islands of the Andaman archipelago, namely, the South Andaman (Mt. Harriet), Rutland, Little Andaman, Havelock Island in the Ritchie’s Archipelago and North Andaman (Saddle Peak) (Fig. 4).

##### Vernacular name.

‘Andaman bush toad’ is proposed as the common English name for this new species, indicating its arboreal habit and restricted distribution as understood currently.

##### Ecological notes.

The new species is often seen on surface of leaves of herbaceous bushes. It is nocturnal and regularly seen year round. It was the third most common anuran in the islands (Harikrishnan and Vasudevan 2015). The high abundance of this species seems to be the result of it occupying a narrow range of distribution and a unique niche of frogs belonging to the Old World tree frog family (Rhacophoridae), which are not known to occur on the Andaman Islands. All other anuran amphibians recorded from these islands are ground-dwelling, with the exception of *Kaloula baleata ghoshi*, which is semi-arboreal, and *Ingerana charlesdarwini*, which is known to use phytotelms for breeding and oviposition (Das 1998). During day time, bush toads were found under leaf litter on the forest floor.

**Figure 5.**
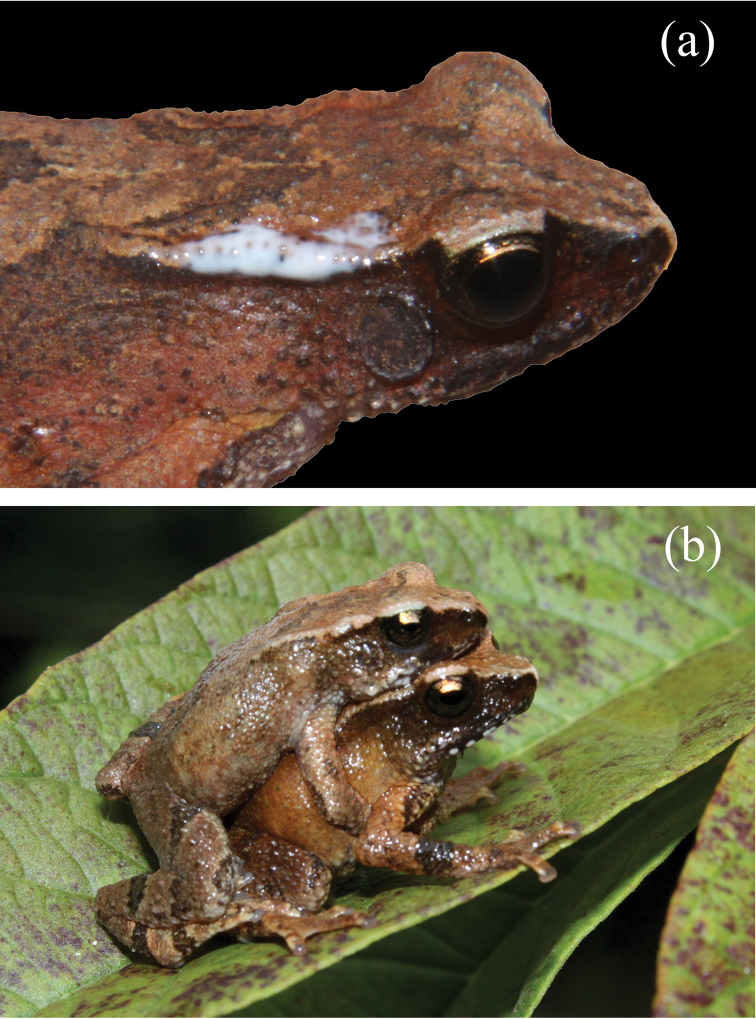
**a** A live, uncollected specimen of *Blythophryne beryet* gen. et sp. n. showing milky white secretion from the parotoid gland **b** Amplecting pair (live, uncollected) of *Blythophryne beryet* gen. et sp. n. showing axillary amplexus.

**Figure 6.**
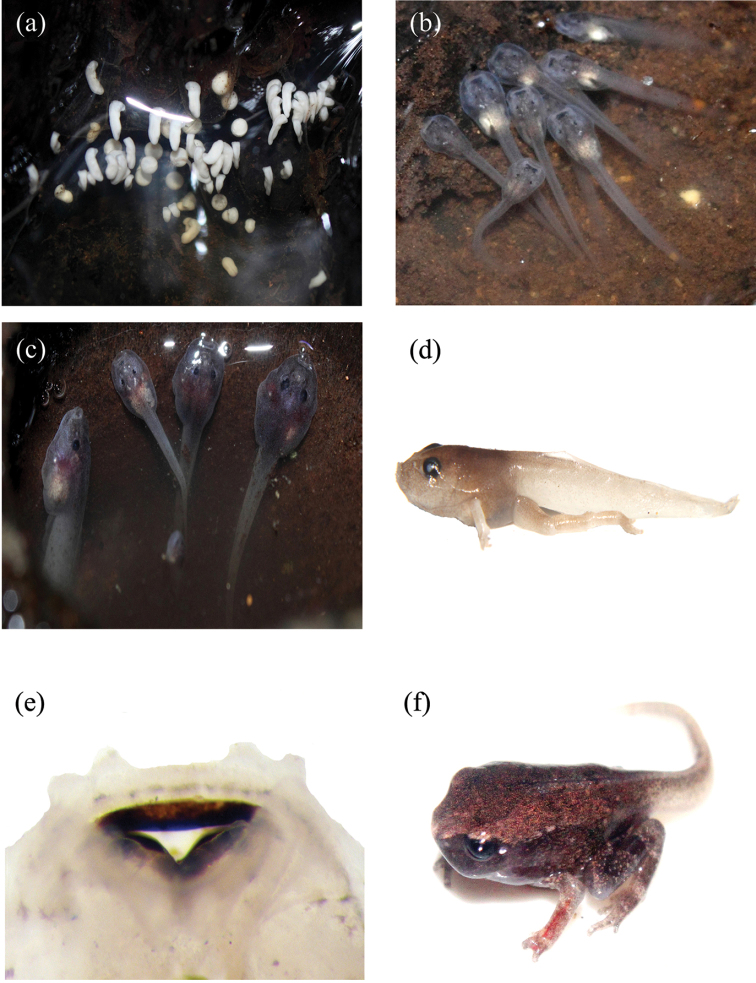
**a** Eggs and hatchling tadpoles of *Blythophryne beryet* gen. et sp. n. **b**, **c** endotrophic larvae of *Blythophryne beryet* gen. et sp. n. showing pale white abdominal yolk **d** Lateral view of a Stage 43 tadpole of *Blythophryne beryet* gen. et sp. n. **e** Oral disc of a Stage 35 larva of *Blythophryne beryet* gen. et sp. n., showing absence of keratodont and the presence of keratinised jaw sheaths **f** a metamorph of *Blythophryne beryet* gen. et sp. n. showing initiation of tail absorption.

The Andaman bush toad emits a white, viscous, pungent smelling secretion from the parotoid glands when handled (Fig. 5a); the secretion seems to be toxic, as other frogs kept within the same bag as one of these toads suffered mortality. Breeding commences in June with the onset of the Southwest Monsoon. Males were observed to call from heights of ca. 1–1.5 m above ground while sitting on leaves of bushes. Amplexus is axillary (Fig. 5b), and females deposit ova in phytotelms, which are tree-holes at a height of about 1–1.5 m above the ground filled with rainwater. Tadpoles develop in these phytotelms. The shrub from which the tadpoles described here were collected, measured 19 cm diameter at breast height, and eggs were found in a depression of 6 cm depth, filled with water up to 3 cm. The tree hole was oval, measuring 5 × 3 cm across (Fig. 6a).

The Andaman bush toad is widely distributed in islands where it occurs, and occupies forested habitats from 29–250 m asl, more common above 100 m asl and rarer at lower altitudes. The forest types in this elevation range include littoral, moist-deciduous, giant evergreen and montane stunted evergreen forests (Champion and Seth 1968).

##### Conservation status.

The Andaman bush toad is known from five islands: North Andaman (Saddle Peak National Park only), South Andaman, Rutland, Havelock (only in a small patch of wet forest towards the south of the island) and Little Andaman. Based on searches carried out using 21 bounded quadrats of 100 m^2^ each in these islands, the new species occurs at densities of 1.1 ± 0.37 toads per 100 m^2^ of forest floor (unpublished data). It is considered ‘Endangered’ based on IUCN Ver. 3.1. Second Edition (IUCN 2014): criteria B.1 - extent of occurrence < 20000 km^2^ and B.1.a - severely fragmented population and known to exist at no more than 10 locations. A large array of invasive fauna in these Islands threatens the population of this toad. Additionally, stochastic events and anthropogenic pressures are potential threats to the species and its habitat.

##### Notes on larval development.

(Fig. 6b–f) The clutch of ova in the phytotelm located in May 2011 at Rutland Island was monitored continuously until complete tadpole transformation. Unpigmented, early-stage larvae were observed on 2^nd^ May 2011. A total of 73 hatchlings presumably from a single clutch could be counted in the phytotelm. Following subsequent rain showers four days later on 6^th^ May, only 25 tadpoles of Stage 20 could be observed, the rest presumably washed out by overflow. At this stage, the tadpoles were translucent and colourless, but speckled with black, with white abdominal yolk region, dorsally positioned eyes and labia visible. On 19^th^ May, i.e., 13 days later, two samples of Stages 30 and 35 were collected and preserved in formalin. Tadpoles of these stages had exposed hind limbs, lacking forelimb buds and were dull purplish-brown in colour, without a dorsal pattern. A week later, on 25^th^ May, the tadpoles that developed into Stages of 41 and 43, were preserved. At these advanced stages, the tadpoles showed developed forelimbs, with expanded discs of fingers, more intense pigmentation on skin, and feeble barred pattern on limbs. The Stage 43 larva is briefly described: mouth positioned anteriorly, with prominent, keratinised pair of jaw sheaths; keratodont absent, eyes and nostrils positioned dorso-laterally (IO 1.46 mm), nostrils much closer to eyes than snout tip. Body depressed, head-body 1.5 times as long as broad (HBL: HBW 1.53), tail almost twice as long as head-body (tL/HBL 1.95) with well-developed caudal musculature. Measurements of the tadpoles are in Table 5.

**Table 5.** Morphometric measurements of tadpoles of *Blythophryne beryet* gen. et sp. n.

Stage	IOL	IND	NED	NSD	SS	SV	BL	TL	MBW	MTH	MTMW	TMH	ODD	VTL	DFH	VFH
**30**	1	0.8	0.4	0.7	3.6	1.6	5	11.1	2.8	2.5	1.1	1.3	1.2	0.7	0.6	0.4
**35**	1.1	1.1	0.4	0.8	5	3.3	6.8	12.4	3.7	3.5	1.3	2	1	1.7	0.9	0.7
**41**	1	1	0.4	0.6	3.9	2.4	6.1	11.8	3.8	2.8	1.3	1.8	1.5	0.9	0.7	0.7
**42**	1.3 (±.20)	1.1	0.4 (±.05)	0.7 (±.10)	NA	NA	7.3 (±.05)	13.6 (±.05)	3.5 (±.05)	3.2 (±.10)	1.8 (±.20)	1.9 (±.15)	1.3 (±.25)	NA	0.9 (±.05)	0.7 (±.05)
**43**	1.5 (±.30)	1.1	0.8 (±.20)	NA	NA	NA	7.0 (±.10)	9.4 (±1.05)	3.8 (±.20)	1.9 (±.35)	1.2 (±.10)	1.6	1.7 (±.10)	NA	0.4 (±.05)	0.3

##### Description of Tadpole

**(Stage 35).** Body tubular in dorsal and ovoid in lateral views, respectively (Fig. 6c). When viewed laterally, body dorsum is flattened and depressed medially; ventrally body slightly flattened at anterior end and convex towards posterior; body length 35% of total length; body attains maximum diameter in region immediately behind eyes. Snout broad and truncate in dorsal and pointed in lateral views, respectively. Eyes large; located and oriented dorso-laterally. Nostrils rounded with elevated rim, located almost midway but closer to eyes than snout, placed linear to eye in dorsal view; internarial distance subequal to interorbital distance. Spiracle sinistral and long with no inner wall; spiracle opening large; tube orientated postero-laterally, opening located approximately at midbody. Distance between spiracle and snout about 60% of body length. Intestinal coils not visible through the belly wall; vent tube medial. Tail tip broadly rounded; musculature linear till 1/3rd length of tail, after which it tapers. Dorsal fin slightly wider than ventral fin, originates posterior to body – tail junction and ventral fin at ventral terminus; both fins run parallel to tail muscle parallel through entire length of tail. Maximum tail height attained at about mid-length. Lateral line faintly visible. No glands observed on outer integument.

Oral disc positioned at terminal portion of body opening antero-ventrally (Fig. 6e); Rostral width of oral disc 27% body width, non-emarginate; entire oral disc visible dorsally; single row of seven to eight large marginal papillae present on lower labium and two to three on lateral corners; none present on upper labium; a single submarginal papilla located at each lateral corners; lower labium larger than upper labium. Denticle rows absent. Jaw sheaths well developed, heavily keratinised. Jaw sheaths completely serrated with minute serrations on lower jaw than upper jaw; suprarostrodont convex medially, longer than wide and lateral process of subequal height through length; infrarostrodont U-shaped.

Measurements (in mm; mean shown without parentheses and standard errors are shown in parentheses): Measurements of the seven tadpoles of various stage of development (Stages 30, 35, 41, 42 and 43) are presented in Table 5.

##### Colour.

In life, dorsally, outer integument brown, with no melanopores. Ventrally, integument translucent but the gut was not visible; throat speckled. Both tail fins transparent with few melanophores. Laterally, tail muscle white with a few brown spots spread mainly at anterior region of tail. A completely transformed metamorph (SVL 10.6 mm; HL 4.23 mm) resembles adult in morphology, with an evident inverted ‘V’ mark on dorsum and transverse crossbars on limbs.

##### Morphological comparisons.

Morphological and osteological characteristics of this new taxon are compared with members of other known Oriental bufonid genera below. The new taxon described here differs from the following known genera thus (only opposing character states in the genera being compared are mentioned):

*Parapelophryne* Fei, Ye & Jiang, 2003: type species– *Nectophryne scalptus* [current name combination: *Parapelophryne scalpta* (Liu and Hu 1973)]: Presence of eight presacral vertebrae and absence of parotoid glands (Fei et al. 2003). The phylogenetic position of this taxon was assessed by Matsui et al. (2015), who found it to be sister taxon to *Bufo japonicus*, thereby providing additional evidence for its distinctness from *Blythophryne* gen. n. described here. Distribution: Hainan, eastern China.

*Pedostibes* Günther, 1875: type species – *Pedostibes tuberculosus* Günther, 1875: Larger adult size (SVL 36.6–38.5 mm), presence of eight presacral vertebrae; short, rounded parotoid glands; tips of fingers dilated into truncated discs; small, numerous pigmented ova laid in strings, as in members of the genus *Duttaphrynus* and exotrophic larvae (Günther 1875, Inger et al. 1984, Fei et al. 2003, Matsui et al. 2007). Currently, the genus *Pedostibes* is represented by five nominal species, which show a disjointed distribution pattern. The westernmost of all, *Pedostibes tuberculosus*, is the type species associated to the generic name (Günther 1875). *Pedostibes kempi* is known from the Garo Hills in Meghalaya, north-east India. Presently, *Pedostibes kempi* is considered congeneric, but differs in having a concealed tympanum. The remaining species, namely, *Pedostibes rugosus*, *Pedostibes hosii* and *Pedostibes everetti* occur in the Indo-Chinese and Indo-Malayan regions (Frost 2014). Bocxlaer et al. (2009), and more recently Ron et al. (2015), in their phylogenetic studies, showed that the genus *Pedostibes*, as currently defined, does not constitute a monophyletic group. According to their study, the type species, *Pedostibes tuberculosus* does not show a close relationship with the south-east Asian *Pedostibes hosii*. On the other hand, they demonstrated that *Pedostibes hosii* is the sister taxon to *Phrynoidis juxtasper*. In addition, the generic placement of *Pedostibes kempi* is also uncertain owing to the inconsistencies in morphological characters associated with this taxon. Hence, resolving the higher level systematic status of the south-east Asian taxa currently allocated to the genus *Pedostibes* will require further study. Distribution: Western Ghats, Indochina, Malay Peninsula.

*Bufoides* Pillai & Yazdani, 1973: type species– *Ansonia meghalayana* [current name combination: *Bufoides meghalayanus* (Yazdani & Chanda, 1971); currently monotypic, but additional, unnamed species recognised; Das et al. 2009]: Larger adult size (mean 42.9 mm, range 37–47 mm), absence of webbing and expanded discs in fingers, hidden tympanum, presence of cranial ridges and large, pigmented ova laid in strings, as in *Duttaphrynus* (Yazdni and Chanda 1972, Pillai and Yazdani 1973, Fei et al. 2003), presence of seven presacral vertebrae, distinguish this taxon from the newly described genus. Distribution: Khasi Hills, Meghalaya, north-east India (Frost 2014). *Pelophryne* Barbour, 1938: type species– *Pelophryne albotaeniata* Barbour, 1938: Presence of coccygeal expansions, absence of parotoid glands; fleshy manus with one phalange free of web and presence of seven (occasionally six) presacral vertebrae, urostyle fused to the sacrum and less number (n ≤ 30) of larger sized yolky eggs (Barbour 1938, Inger 1954, 1966; Matsui et al. 2007). Distribution: eastern Asia, Sundaland and the Philippines (Frost 2011).

*Sabahphrynus* Matsui, Yambun & Sudin, 2007: type species– *Nectophryne maculata* [current name combination: *Sabahphrynus maculatus* (Mocquard, 1890)]: Larger adult size (41.21 ± 2.5, 30.4–52.6), presence of eight presacral vertebrae, absence of tympanum and parotoid glands, absence of webbing between the fingers, over 50 eggs/ovary and absence of an external vocal sac in males (Matsui et al. 2007). Distribution: endemic to Borneo (Frost 2014).

*Duttaphrynus* Frost, Grant, Faivovich, Bain, Haas, Haddad, de Sá, Channing, Wilkinson, Donnellan, Raxworthy, Campbell, Blotto, Moler, Drewes, Nussbaum, Lynch, Green & Wheeler, 2006: type species– *Bufo melanostictus* [current name combination: *Duttaphrynus melanostictus* (Schneider, 1799)]: Large adult size (mean SVL 43.7 mm), presence of eight presacral vertebrae, presence of keratinised cephalic ridges in some species, presence of large, keratinised warts on the dorsum, absence of expanded discs in finger and toe tips, absence of webbing between the fingers, numerous black pigmented ova laid in long, continuous strings, exotrophic larvae and terrestrial habit (Dubois and Ohler 1999, Manamendra-Arachchi and Pethiyagoda 1998). Particularly, the nomen *Bufo camortensis* (holotype – ZSI A 6955) erected for a species that is currently considered to represent *Duttaphrynus melanostictus* differs from the new taxon described here by its considerably large adult size [SVL – 67 mm (*vs*. much smaller mean adult size of 24 mm in *Blythophryne* gen. n.), presence of keratinised cephalic ridges and glandular tubercles on the body (vs. absent in *Blythophryne* gen. n.), absence of webbing between the fingers and dilated terminal discs in the digits (vs. present in *Blythophryne* gen. n.). Distribution: East Africa through the Middle East, India, Indochina, east to the Sundas till Bali (Frost 2014).

*Ansonia* Stoliczka, 1870: type species – *Ansonia penangensis* Stoliczka, 1870: small to medium adult size (35–40 mm), absence of (or rudimentary) webbing between the fingers, presence of eight presacral vertebrae, absence of dilations in finger and toe tips, absence of parotoid glands, exotropic larvae with prominent oral discs and torrential stream dwelling habit (Inger 1960, Matsui et al. 2010). Distribution: Indo-Malayan region and the Philippines (Frost 2014).

*Adenomus* Cope, 1861: type species– *Adenomus badioflavus* Cope, 1861, a junior synonym of *Bufo kelaartii* [current name combination: *Adenomus kelaarti* (Günther, 1858)]: The genus *Adenomus* was resurrected from the synonymy of ‘*Bufo*’ by Manamendra-Arachchi and Pethiyagoda (1998) to accommodate members of the ‘Bufo’ kelaarti group, characterised by smooth finger edges; differing from the new taxon described here by its larger adult size (mean SVL 38.4 mm), presence of seven presacral vertebrae, absence of sheath-like webbing between fingers, absence of expanded discs at digit tips, presence of cranial ridges and indistinct tympanum (in *Adenomus kelaarti*), terrestrial habit, pronounced sexual size dimorphism and unpigmented ova laid in long, continuous strings as in *Duttaphrynus* (Manamendra-Arachchi and Pethiyagoda 1998; Haas 1999; Meegaskumbura et al. 2015). Distribution: endemic to Sri Lanka (Frost 2014).

*Ghatophryne* Biju, Bocxlaer, Giri, Loader & Bossuyt, 2009: type species– *Ansonia ornata* [current name combination: *Ghatophryne ornata* (Günther, 1876)]: larger adult size (up to 35 mm SVL), characteristic reddish dorsal and ventral colouration, absence of parotoid glands, absence of webbing between the fingers, finger tips not dilated to discs and torrential stream dwelling habit (Biju et al. 2009). Distribution: Central Western Ghats in the states of Kerala and Karnataka (Frost 2014).

*Xanthophryne* Biju Bocxlaer, Giri, Loader & Bossuyt, 2009: type species– *Bufo koynaensis* [current name combination: *Xanthophryne koynaensis* (Soman, 1963)]: Larger adult size (up to 35.3 mm SVL), presence of characteristic chrome yellow patches along the flanks and sides of the abdomen, indistinct tympanum, weak, rounded parotoid glands, absence of webbing in fingers and discs in toes and fingers; large, pigmented ova laid in stagnant puddles on the ground (Biju et al. 2009). Distribution: Known only from Northern Western Ghats in Maharashtra, India (Frost 2014).

*Leptophryne* Fitzinger, 1843: type species – *Bufo cruentatus* [current name combination: *Leptophryne cruentata* (Tschudi, 1838)]: Dubois (1982) resurrected the genus *Leptophryne* Fitzinger, 1843 as the senior synonym of *Cacophryne* Davis, 1935, which currently comprises two species – *Leptophryne borbonica* (Tschudi, 1838) and *Leptophryne cruentata* (Tschudi, 1838). Presence of eight presacral vertebrae; firmisternal pectoral girdle; elongate subarticular tubercles near the base of each toe, numerous pigmented eggs and exotrophic larvae (Fei et al. 2003) distinguish it from *Blythophryne beryet* gen. et sp. n. Distribution: Sundaland (Frost 2014).

*Pseudobufo* Tschudi, 1838: type species – *Pseudobufo subasper* Tschudi, 1838: Large body size, stout habitus; presence of seven presacral vertebrae (vs. six in *Blythophryne* gen. n.) completely (to the tip of Toe IV) webbed feet (vs. incomplete toe webbing in *Blythophryne beryet* gen. et sp. n.), fingers basally webbed; parotoid glands absent; dorsal, lateral and ventral skin surfaces with fine spinules, dorsoventrally depressed body with large, round warts and dorsally positioned nostrils (vs. lateral) distinguish it from the new genus described here (Fei et al. 2003; Inger and Stuebing 2005). Distribution: Sundaland.

*Ingerophrynus* Frost, Grant, Faivovich, Bain, Haas, Haddad, de Sá, Channing, Wilkinson, Donnellan, Raxworthy, Campbell, Blotto, Moler, Drewes, Nussbaum, Lynch, Green & Wheeler, 2006: type species– *Bufo biporcatus* [current name combination: *Ingerophrynus biporcatus* (Gravenhorst, 1829)]: Presence of seven presacral vertebrae (vs. six); absence of lateral dilations in the digit tips (vs. present); absence of webbing between the fingers (vs. present) and endotrophic (vs. exotrophic) larvae distinguish *Blythophryne beryet* gen. et sp. n. from *Ingerophrynus*. Distribution: Southern Yunnan, Indochina, the Malay Peninsula, the islands of Indo-Malaya, and Philippines.

*Phrynoidis* Fitzinger, 1843: type species – *Bufo asper* [current name combination: *Phrynoidis asper* (Gravenhorst, 1829)]: Large adult size (up to 100 mm SVL) presence of an omosternum, (vs. absent); presence of seven presacral vertebrae (vs. six); absence of lateral dilations of digit tips (vs. present) and exotrophic (vs. endotrophic) larvae distinguish this genus from the new genus *Blythophryne* gen. n. Distribution: Myanmar through western and peninsular Thailand, the Malay Peninsula, Sumatra, Borneo, and Java.

Apart from the above bufonid genera known from Oriental Asia, the new taxon described herein differs from the following central-west African genera:

*Nectophryne* Buchholz & Peters, 1875: type species – *Nectophryne afra* Buchholz & Peters, 1875 by the presence of eight presacral vertebrae (vs. six in *Blythophryne beryet* gen. et sp. n.); presence of lamelliform subdigital pads – a character unique to *Nectophryne* which is absent in the new taxon described here. Oriental forms including members of the genera *Pedostibes* and *Pelophryne* were attributed to *Nectophryne* earlier (Boulenger 1892, 1896, 1919), until Barbour (1938) redefined these genera.

*Nectophrynoides* Noble, 1926: type species – *Nectophryne tornieri* [current name combination: *Nectophrynoides tornieri* (Roux, 1906)]: The comparisons made here are restricted to the type species of *Nectophrynoides* because the genus is poorly defined and is composed of representatives with a broad spectrum of morphological and developmental characteristics. Though unique among bufonids in possessing an omosternum and a direct developmental mode (in *Nectophrynoides viviparus*), members of this genus are poorly diagnosed with respect to other genera (Menegon et al. 2004). Larger adult size (SVL 21–30 mm), presence of expanded, truncate fingertips (vs. expanded and curved in *Blythophryne beryet* gen. et sp. n.), presence of eight presacral vertebrae (vs. 6 in *Blythophryne beryet* gen. et sp. n.) however, distinguish *Nectophrynoides* from the new taxon described here (see Tihen 1960; Menegon et al. 2004; Harper et al. 2010).

##### Molecular phylogeny.

**Figure 7.**
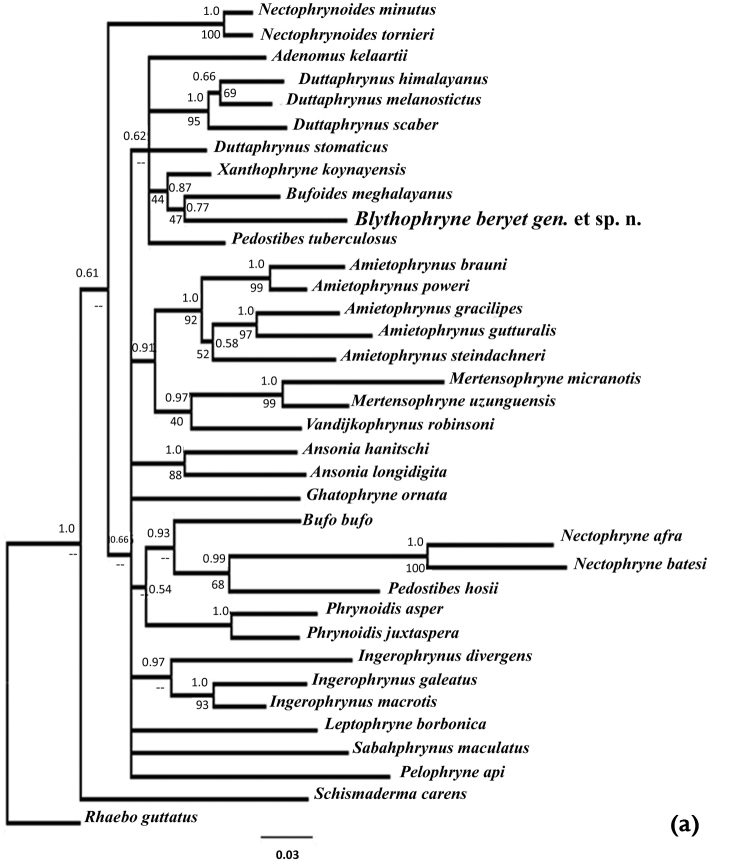

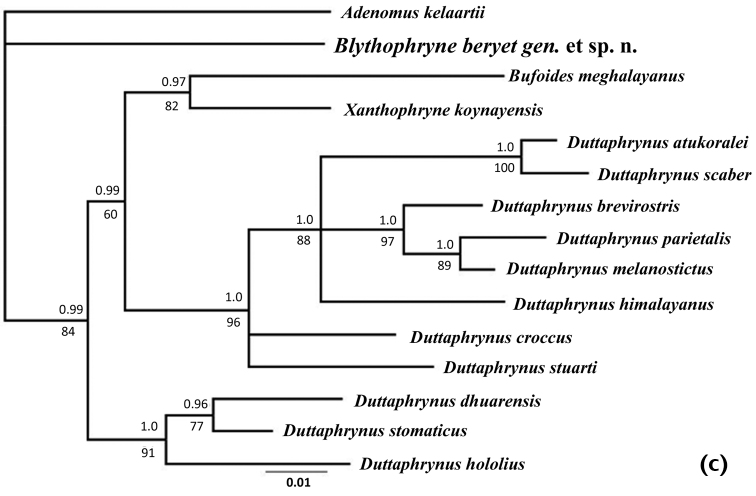
Phylogenetic position of *Blythophryne beryet* gen. et sp. n., inferred from concatenated partial 12S and 16S rDNA sequences. The posterior probabilities for Bayesian Inference (BI) and the bootstrap support values for the ML are given as (BI /ML) above and below the branch nodes. **a** The tree was generated using 36 species related to 21 genera, and was rooted using *Rhabeo gutattus* as outgroup **b** the subclade containing the Indian and Sri Lankan toads (7 genera, 17 species) rooted using *Ghatophryne ornata* as outgroup; and **c** the subclade containing the Indian and Sri Lankan toads (5 genera, 15 species) rooted using *Adenomus kelaartii* as outgroup.

Multiple sequence alignment of the 16S homologous regions resulted in 498 conserved sites and 246 parsimoniously informative sites. In the phylogenetic analysis using both Maximum likelihood and Bayesian inference, the focal taxon showed a unique taxonomic position. The phylograms of both inference methods were similar (Fig. 7). *Bufoides meghalayanus* was found to be the closest taxon to the focal species, *Blythophryne beryet* gen. et sp. n. in the tree generated using 36 species from Asia and Africa but with relatively low support (Fig. 7a). However, when analysed with more of the Asian toads, it clearly separates out from species of *Duttaphrynus*, as well as, those of *Xanthophryne* and Bufoides (Fig. 7b, c). The average within-genus pairwise K2p distances at the partial 16S rRNA gene for all the described genera considered under this study was 0.0642, with 99% confidence interval (CI) of 0.0512–0.0687 (Table 6). The average pairwise k2p distance of the focal species with all other taxa at partial 16S rRNA gene considered here was 0.103, with a 99% CI of 0.096–0.113, strongly supporting its distinctiveness and unique phylogenetic position within the Bufonids. Similarly, for partial 12S rRNA gene, the average within-genus pairwise K2p distances for all described genera was 0.0495, with the 99% CI of 0.0387–0.0603. The average pairwise k2p distance of the focal species with all other taxa at partial 12S rRNA gene was 0.0783, with a 99% CI of 0.072–0.085. Both tree-based and distance-based analyses clearly indicate the uniqueness of its phylogenetic position. Thus, the rDNA typing strongly suggest the new taxon as a candidate to be named as a new genus/species.

**Table 6.** The average uncorrected K2p distance estimates across genera of Bufonidae based on partial 16S (lower triangular matrix) and 12S (upper triangular matrix) rDNA sequences, calculated using MEGA6.06; within genus K2p distances are given along the diagonal (left value for 16S and right value for 12S).

		*16S/12S k2p uncorrected pair-wise distance estimates*																				
Taxa (Genus*/Species)		1	2	3	4	5	6	7	8	9	10	11	12	13	14	15	16	17	18	19	20	21
1	*Amietophrynus*	**0.068/0.044**	0.060	0.049	0.072	0.067	0.094	0.066	0.059	0.074	0.071	0.066	0.049	0.077	0.043	0.100	0.103	0.067	0.063	0.043	0.037	0.071
2	*Ansonia*	0.106	**0.066/0.060**	0.061	0.086	0.083	0.115	0.079	0.066	0.081	0.073	0.065	0.071	0.104	0.061	0.114	0.097	0.067	0.067	0.055	0.063	0.085
3	*Duttaphrynus*	0.092	0.089	**0.049/ 0.038**	0.070	0.063	0.099	0.067	0.060	0.065	0.076	0.065	0.054	0.084	0.045	0.098	0.099	0.074	0.054	0.045	0.049	0.077
4	*Ingerophrynus*	0.090	0.084	0.074	**0.056/0.077**	0.077	0.102	0.075	0.082	0.073	0.085	0.071	0.081	0.093	0.065	0.113	0.111	0.078	0.071	0.062	0.075	0.085
5	*Mertensophryne*	0.095	0.100	0.099	0.090	**0.059/ 0.061**	0.098	0.079	0.066	0.075	0.083	0.075	0.079	0.094	0.067	0.108	0.114	0.083	0.079	0.059	0.065	0.073
6	*Nectophryne*	0.143	0.141	0.127	0.139	0.140	**0.089/0.049**	0.092	0.095	0.098	0.107	0.093	0.098	0.108	0.073	0.146	0.136	0.102	0.083	0.090	0.102	0.081
7	*Nectophrynoides*	0.097	0.091	0.077	0.082	0.099	0.132	**0.021/0.015**	0.068	0.084	0.069	0.069	0.069	0.085	0.051	0.119	0.089	0.061	0.077	0.034	0.077	0.069
8	*Pedostibes*	0.091	0.086	0.066	0.083	0.104	0.115	0.074	**0.076/0.065**	0.075	0.071	0.067	0.063	0.085	0.051	0.130	0.107	0.068	0.069	0.048	0.063	0.071
9	*Phrynoidis*	0.086	0.084	0.073	0.082	0.102	0.135	0.080	0.080	**0.039/0.085**	0.089	0.079	0.091	0.108	0.063	0.123	0.119	0.085	0.069	0.071	0.085	0.081
10	*Ghatophryne ornata*	0.092	0.104	0.075	0.086	0.103	0.148	0.088	0.083	0.087	**n/a**	0.061	0.081	0.102	0.053	0.124	0.097	0.081	0.077	0.057	0.065	0.086
11	*Leptophryne borbonica*	0.103	0.107	0.095	0.096	0.117	0.121	0.099	0.082	0.099	0.092	**n/a**	0.073	0.085	0.045	0.106	0.085	0.069	0.053	0.061	0.061	0.069
12	*Vandijkophrynus robinsoni*	0.083	0.102	0.063	0.071	0.082	0.132	0.077	0.077	0.073	0.064	0.102	**n/a**	0.072	0.053	0.110	0.097	0.073	0.072	0.037	0.049	0.085
13	*Schismaderma carens*	0.096	0.099	0.076	0.088	0.086	0.132	0.082	0.079	0.095	0.097	0.107	0.092	**n/a**	0.089	0.110	0.110	0.089	0.069	0.077	0.081	0.094
14	*Bufo bufo*	0.102	0.113	0.078	0.107	0.116	0.134	0.085	0.088	0.082	0.087	0.105	0.076	0.105	**n/a**	0.110	0.085	0.057	0.057	0.034	0.045	0.057
15	*Sabahphrynus maculatus*	0.092	0.085	0.077	0.071	0.097	0.124	0.093	0.079	0.081	0.082	0.093	0.087	0.082	0.104	**n/a**	0.145	0.114	0.110	0.102	0.106	0.123
16	*Pelophryne api*	0.108	0.105	0.102	0.092	0.100	0.155	0.106	0.097	0.106	0.100	0.113	0.100	0.122	0.126	0.112	**n/a**	0.089	0.093	0.068	0.101	0.093
17	*Bufoides meghalayanus*	0.088	0.091	0.055	0.080	0.109	0.133	0.073	0.064	0.069	0.078	0.095	0.062	0.087	0.071	0.087	0.105	**n/a**	0.073	0.041	0.069	0.069
18	*Adenomus kelaartii*	0.091	0.091	0.065	0.084	0.107	0.136	0.070	0.071	0.070	0.066	0.098	0.059	0.090	0.083	0.085	0.103	0.062	**n/a**	0.061	0.069	0.065
19	*Xanthophryne koynayensis*	0.084	0.078	0.054	0.070	0.098	0.129	0.072	0.056	0.077	0.073	0.087	0.059	0.082	0.078	0.078	0.083	0.039	0.057	**n/a**	0.049	0.049
20	*Rhaebo guttatus*	0.111	0.085	0.092	0.088	0.104	0.136	0.089	0.083	0.086	0.087	0.105	0.092	0.088	0.114	0.094	0.112	0.092	0.085	0.075	**n/a**	0.086
21	***Blythophryne beryet*** **gen. et sp. n.**	0.103	0.119	0.089	0.098	0.118	0.165	0.098	0.102	0.088	0.099	0.112	0.092	0.101	0.105	0.106	0.112	0.080	0.082	0.075	0.109	**n/a**

# The data in the first nine rows for samples ‘1’ to ‘9’are average k2p estimates (Intra-/inter species) for all the species of the indicated genus considered in the study; these are as follow: Taxa-1: *Amietophrynus* : *Amietophrynus brauni*, *Amietophrynus poweri*, *Amietophrynus gracilipes*, *Amietophrynus gutturalis*, *Amietophrynus steindachneri* Taxa-2: *Ansonia* : *Ansonia hanitschi*, *Ansonia longidigita* Taxa-3: *Duttaphrynus* : *Duttaphrynus himalayanus*, *Duttaphrynus melanostictus*, *Duttaphrynus scaber*, *Duttaphrynus stomaticus* Taxa-4: *Ingerophrynus* : *Ingerophrynus divergens*, *Ingerophrynus macrotis*, *Ingerophrynus galeatus* Taxa-5: *Mertensophryne* : *Mertensophryne micranotis*, *Mertensophryne uzunguensis* Taxa-6: *Nectophryne* : *Nectophryne afra*, *Nectophryne batesi* Taxa-7: *Nectophrynoides* : *Nectophrynoides minutus*, *Nectophryne tornieri* Taxa-8: *Pedostibes* : *Pedostibes hosii*, *Pedostibes tuberculosus* Taxa-9: *Phrynoidis* : *Phrynoidis asper*, *Phrynoidis juxtasper*

## Discussion

The small-sized bush toad described here is an interesting new find from the Andaman Islands, in the Bay of Bengal, Republic of India. It has a number of unique external morphological and skeletal characters, in comparison to known Oriental and other relevant bufonid genera. Its distinctiveness and unique taxonomic position (warranting the erection of a monotypic genus), is also robustly supported by phylogenetic reconstruction carried out using partial 16S and 12S gene sequences and showing its position relative to other Asian and African bufonids (Pramuk et al. 2008; Van Bocxlaer et al. 2009, 2010; Matsui et al. 2007). Much of the rapid radiation and diversification of toads happened during the Paleogene, and show short intermodal distances (Pramuk et al. 2008). The phylogenetic inference obtained in the present study is concordant with those of the earlier studies.

**Biogeographic remarks.** Bufonidae is a species-rich family, with nearly cosmopolitan distribution around the globe (Frost 2014). Pramuk et al. (2008) suggests a post-Gondwanan, South American origin of the family, and a rapid diversification and dispersal across the globe, and a return to South America within a short span of 80 million years. They hypothesised overland dispersal routes for both out-of and into-South America. While this explains the possible routes of dispersal and diversification of bufonids across the continental mainland, the routes of diversification of the Bufonidae on islands is unclear, including evolution of endemic bufonid lineages on Sri Lanka, insular south-east Asia and the Andaman Islands.

The herpetofauna of Andaman and Nicobar Islands are considered to be of either Indo-Chinese or Indo-Malayan affinities (Das 1999). While it is hypothesised that the Nicobar Islands are of volcanic origin, most of the Andaman Islands are uplift of submerged landmass (Krishnan 1961). Exchange of biota would have been facilitated via either a physical connection of the islands to the mainland during lowering of sea level (Rodolfo 1969) or through trans-oceanic or other forms of across-water dispersal, especially for the Nicobar archipelago. There are records of long-distance overseas dispersal routes, which could be the only possible route for certain endemic taxa of the archipelago, such as the Andaman Day Gecko, *Phelsuma andamanense* (see Austin et al. 2004). Amphibians, although generally considered intolerant to salinity, have also been known to show long-distance, overseas dispersal (e.g., Vences et al. 2003).

The submerged chain of mountains referred to as the “Burma arc” was formed at the same time as the main Himalayan chain, during the late Cretaceous (Krishnan 1961). The occurrence of a distinct lineage prompts us to propose the following explanations: (i) overland dispersal when the Islands were connected to the mainland due to lowering of sea level; (ii) trans-oceanic dispersal; (iii) relic lineage surviving in the Islands due to a vicariant event that might have occurred during Cretaceous by isolation in on mountain tops on the “Burma arc”. While there are also records of long-distance overseas dispersal into the Islands, such as Andaman day Gecko, *Phelsuma andamanense* (see Austin et al. 2004) and in frogs (e.g. Vences et al. 2003), evidence for the other hypotheses are clearly not available at present. Scanty geological data and poor sampling of toad lineages in the mountains of Myanmar that precludes unambiguous molecular dating of sister lineages, also make it difficult to infer the biogeographic affinities of the Andaman bush toad at present.

The new taxon described here is characterised with a small adult body size, semi-arboreality high specificity for larval microhabitat niche, absence of inguinal fat bodies, relatively low number of mid-sized ova and a narrow distributional range. Further, it seems to be an exception in possessing parotoid glands, which was a character associated with widely distributed bufonid species (Bocxlaer et al. 2010), and presumably relate to reduction of predation via development of specialised glands for storage of dietary-sequestered toxins.

Likewise, the larvae of this new taxon with a moderate, intermediate clutch size and a high specificity towards the site of oviposition (i.e., phytotelms) explain its limited range of distribution as currently understood. Further studies in the Andaman archipelago are needed to understand the identity and origins of its fauna.

## Supplementary Material

XML Treatment for Blythophryne

## Appendix 1

List of comparative material of Oriental and other relevant members of the Bufonidae examined.

*Adenomus ‘dasi*’ WHT 2267–69; *Adenomus kandianus* BMNH 1947.2.62–63; *Adenomus kelaartii* MNHN 140.0; WHT 1447; WHT 1451; *Ansonia guibei* UNIMAS 7746; *Ansonia hanitschi* UNIMAS 8050; *Ansonia longidigita* UNIMAS 7925–26; *Ansonia minuta* UNIMAS 7427; *Ansonia muleleri* FMNH 96125 (cleared and stained); *Ansonia penangensis* USNM 216034 (radiograph), ZSIC 2717–18; 3585–61; *Ansonia spinulifer* UNIMAS 7580; *Bufoides meghalayanus* WII uncatalogued (cleared and stained) ZSIC A6969–70; *Duttaphrynus melanostictus* WII 38.6.92 (cleared and stained); WHT 2276; UNIMAS 9313, UNIMAS 9349; *Duttaphrynus olivaceus* ZSIC 3523–25; *Duttaphrynus silentvalleyensis* ZSIM/SRS VA/77; *Duttaphrynus stuarti* ZSIC 19958; *Ghatophryne rubigina* ZSIM/SRS VA/775; *Ingerophrynus divergens* FMNH 138867 (cleared and stained); UNIMAS 7943; *Ingerophrynus kumquat* ZRC 1.3137–42; 1.3584; *Ingerophrynus parvus* ZSI 15196–97; *Ingerophrynus quadriporcatus* UNIMAS 9433; *Leptophryne borbonica* FMNH 185792 (cleared and stained); UNIMAS 9055; *Nectophryne afra* ZMB8472 (holotype); MCZ A2607 (radiographs); *Pedostibes hosii* FMNH 77369 (cleared and stained); UNIMAS 8434; UNIMAS 8972; *Pedostibes tuberculosus* WII 38.6.91 (cleared and stained); *Pedostibes kempi* ZSI 18481 (syntype); *Pelophryne albotaineata* MCZ A–23291 (holotype; radiograph); *Pelophryne linanitensis* ZRC 1.11906–10; *Pelophryne misera* UNIMAS 8053; *Pelophryne murudensis* ZRC 1.11902–905; *Pelophryne signata* UNIMAS 7589, UNIMAS 7930, UNIMAS 7931; *Phrynoidis asper* FMNH 219718 (cleared and stained); UNIMAS 7874; UNIMAS 9432; *Pseudobufo subasper* FRIM uncat., USNM 313624, MCZ A 19579; *Sabahphrynus maculatus* MNHN P1899–267 (lectotype; radiograph); *Xanthophryne koynayensis* BNHM 377; ZSIC A1784; ZSIM/SRS VA/775.
